# Pilot Study for Deciphering Post-Translational Modifications and Proteoforms of Tau Protein by Capillary Electrophoresis-Mass Spectrometry

**DOI:** 10.1021/acs.jproteome.4c00587

**Published:** 2024-09-27

**Authors:** Fei Fang, Tian Xu, Hsiao-Tien Chien Hagar, Stacy Hovde, Min-Hao Kuo, Liangliang Sun

**Affiliations:** † Department of Chemistry, Michigan State University, 578 S Shaw Lane, East Lansing, Michigan 48824, United States; ‡ Department of Biochemistry and Molecular Biology, Michigan State University, 603 Wilson Road, Room 401, East Lansing, Michigan 48824, United States

**Keywords:** tau protein, Alzheimer’s disease, capillary electrophoresis-mass spectrometry, phosphorylation, post-translational modification, proteoform

## Abstract

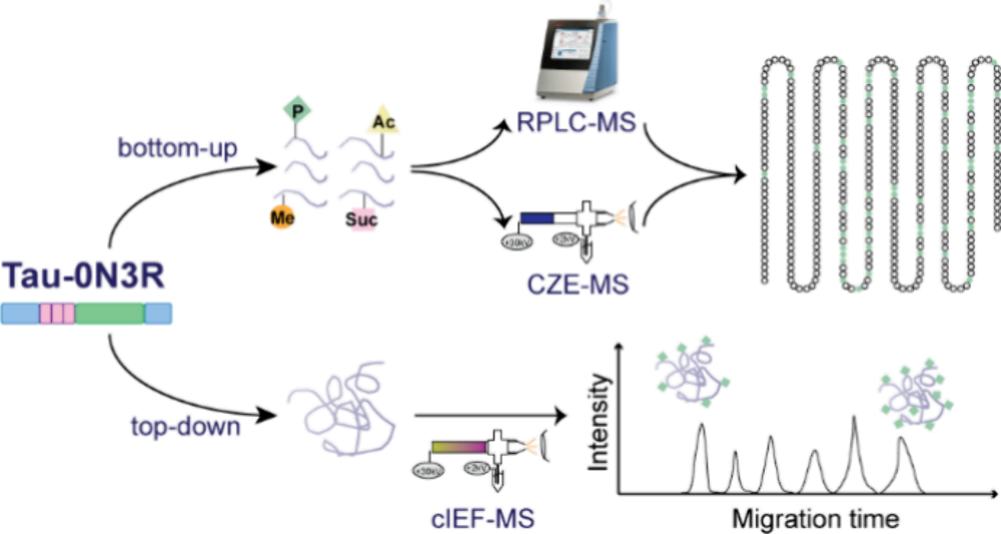

Abnormal accumulation of tau protein in the brain is one pathological hallmark of Alzheimer′s disease (AD). Many tau protein post-translational modifications (PTMs) are associated with the development of AD, such as phosphorylation, acetylation, and methylation. Therefore, a complete picture of the PTM landscape of tau is critical for understanding the molecular mechanisms of AD progression. Here, we offered a pilot study of combining two complementary analytical techniques, capillary zone electrophoresis (CZE)-tandem mass spectrometry (MS/MS) and reversed-phase liquid chromatography (RPLC)-MS/MS, for bottom-up proteomics of recombinant human tau-0N3R. We identified 50 phosphorylation sites of tau-0N3R in total, which is about 25% higher than that from RPLC-MS/MS alone. CZE-MS/MS provided more PTM sites (i.e., phosphorylation) and modified peptides of tau-0N3R than RPLC-MS/MS, and its predicted electrophoretic mobility helped improve the confidence of the identified modified peptides. We developed a highly efficient capillary isoelectric focusing (cIEF)-MS technique to offer a bird’s-eye view of tau-0N3R proteoforms, with 11 putative tau-0N3R proteoforms carrying up to nine phosphorylation sites and lower pI values from more phosphorylated proteoforms detected. Interestingly, under native-like cIEF-MS conditions, we observed three putative tau-0N3R dimers carrying phosphate groups. The findings demonstrate that CE-MS is a valuable analytical technique for the characterization of tau PTMs, proteoforms, and even oligomerization.

## Introduction

Over 20 adult neurodegenerative diseases, such as Alzheimer’s disease (AD), are characterized by the dysfunction and accumulation of tau protein.^1−4^ However, the molecular mechanisms that induce tau misfolding and aggregation in neurodegenerative disorders remain elusive.^5^ Tau protein post-translational modifications (PTMs) are associated with the development of AD, such as phosphorylation, acetylation, and methylation.^6−9^ Therefore, a complete picture of the PTM landscape of tau is critical for understanding the roles played by tau protein in modulating AD progression.

The analytical methods for tau PTM characterization include neuroimaging, biosensing, immunoassay, and mass spectrometry (MS) techniques.^10,11^ Among them, immunoassay, which is simple to implement and provides semiquantitative information, has been the workhorse for the identification and quantification of tau or phosphorylated tau (p-tau).^12^ However, the antibody-based targeted approach impedes the discovery of new PTMs that could be critical for AD progression.

Emerging MS-based proteomics approaches are powerful alternative methods used to characterize PTMs. In particular, reversed-phase liquid chromatography-tandem MS (RPLC-MS/MS) has been widely used for the characterization of tau PTMs.^13^ For example, Steen and co-workers developed an MS-based assay, Full-Length Expressed stable Isotope-labeled Tau (FLEXITau), with 55 phosphosites, 17 ubiquitination sites, 19 acetylation sites, and 4 methylation sites mapped along the 2N4R tau protein from 91 brain tissues.^7,14,15^ With a targeted parallel reaction monitoring strategy, Barthélemy et al. detected 29 distinct phosphorylated tau sites in the brain and cerebrospinal fluid in humans.^16^

Due to its high separation performance and high detection sensitivity, capillary zone electrophoresis (CZE)-MS/MS is becoming an attractive tool for monitoring highly modified samples, such as histone proteoforms^17−20^ and post-translationally modified peptides.^21^ With CZE separating analytes based on their electrophoretic mobilities corresponding to their charge-to-size ratios, not their hydrophobic nature, the combination of RPLC-MS/MS and CZE-MS/MS could impressively expand the PTM coverage.^21,22^ However, to the best of our knowledge, no investigators have reported on tau PTM analysis with the combination of CZE-MS/MS and RPLC-MS/MS.

In this work, for the first time, CZE-MS/MS was investigated for the characterization of tau PTMs and was compared with RPLC-MS/MS in terms of identified modified peptides. We also applied capillary isoelectric focusing (cIEF)-MS to measure the intact tau proteins for a bird’s-eye view of tau proteoforms carrying combinations of PTMs.

## Experimental Section

### Chemicals

All chemicals were purchased from Sigma-Aldrich (St. Louis, MO), unless otherwise specified. LC-MS grade solvents, including water, acetonitrile (ACN), methanol, formic acid (FA), and acetic acid, were purchased from Fisher Scientific (Pittsburgh, PA). Acrylamide was purchased from Acros Organics (NJ, USA). Fused silica capillaries (50 μm i.d./360 μm o.d.) were purchased from Polymicro Technologies (Phoenix, AZ).

### p-tau (0N3R) Expression in *E. coli* Cells and Purification

The designing principle of plasmids and the procedures for preparing PIMAX phosphorylated tau (p-tau) are based on our previous work.^8,9,23^ The expression approach can produce specific tau isoforms (e.g., 0N3R) with high purity.^9^ Briefly, cyclin-dependent kinase 5 (CDK5) and the human 0N3R tau isoform were brought to the proximity by heterodimerization of Fos and Jun leucine zipper domains. In addition, a separate plasmid was transformed into the same *E. coli* strain to supply the cyclin protein p25. The overnight culture of BL21-CodonPlus cells harboring PIMAX tau plasmid was diluted to 0.03 OD600 and grown at 37 °C with shaking at 230 rpm until reaching 0.3–0.5 OD600. Then, the protein expression was induced at 37 °C for 2 h with 0.2 mM IPTG, and the bacterial pellet was collected by centrifugation. The pellet from 1 L of induction was suspended with 10 mL of lysis buffer (20 mM Tris, pH 5.8, 100 mM NaCl) with 1 mg/mL lysozyme, 1 mM PMSF, 0.2 mM orthovanadate, and 1 tablet of Roche protease inhibitor and incubated for 45 min at room temperature. The suspension was sonicated afterward (Branson Digital Sonifier 450; 30% amplitude; total process time: 3 min; pulse-ON time: 5 s; pulse-OFF time: 5 s). The sonication supernatant was boiled for 30 min in boiling water after centrifugation at 17,000 *g* for 30 min at 4 °C, and the boiled sample was centrifuged afterward. The boiled supernatant was digested with purified recombinant TEV protease in the ratio of 1 OD280 per 100 OD280 of the sample in the presence of 0.5 mM dithiothreitol (DTT) and 1 mM EDTA (ethylenediaminetetraacetic acid). The reaction mixture was incubated at 4 °C overnight. The digestion mixture was centrifuged at 17,000 *g* for 30 min at 4 °C, and the supernatant was transferred to another tube and concentrated with an Amicon spin column (Amicon Centrifugal Filter Unit, Ultra-15, 30 K) at 5000 *g* at 4 °C. The buffer was changed to storage buffer (20 mM Tris-HCl pH 7.4, 100 mM NaCl) in the meantime. The concentrated sample was supplemented with 10% glycerol (v/v) and stored at −80 °C for further usage.

### Preparation of Human p-tau (0N3R) Protein for Bottom-Up Proteomics

The protein digestion was performed with the filter-aided sample preparation protocol with minor modifications.^24^ 20 μg of the p-tau protein sample was loaded onto a Microcon 30 kDa molecular weight cutoff centrifugal filter, and the buffers were exchanged to 50 μL of 8 M urea dissolved in 50 mM ammonium bicarbonate (NH_4_HCO_3_). The filter unit including the samples was subjected to denaturation and reduction by incubating at 37 °C for 30 min with the addition of 10 mM DTT. Followed by alkylation with 25 mM iodoacetamide for 30 min in the dark, the filter unit was washed with 50 mM NH_4_HCO_3_ four times at 14,000 *g*. The samples were suspended in 50 μL of 50 mM NH_4_HCO_3_ after cleanup. Afterward, trypsin was added to the sample at a mass ratio of 1:30 for the sample digestion. The filter units were further incubated at 37 °C for 4 h. The peptides from protein digestion were collected by centrifugation at 14,000 *g* for 15 min. To reduce the sample loss due to incomplete digestion, 20 μL of 50 mM NH_4_HCO_3_ buffer was added to the filter unit and further incubated at 37 °C overnight. The flow-through was collected by centrifugation and combined with the corresponding peptide samples from 4 h of digestion.

### CZE-MS/MS and RPLC-MS/MS for Bottom-Up Proteomics

The sample digest of p-tau was analyzed by CZE-MS/MS and RPLC-MS/MS. A CESI 8000 Plus CE system (Sciex) was coupled to a Q Exactive HF mass spectrometer (Thermo Fisher Scientific) via a commercialized electrokinetically pumped sheath-flow CE-MS nanospray interface (EMASS-II, CMP Scientific) for CZE-MS/MS of p-tau peptides.^25,26^ A 1 m linear polyacrylamide (LPA)-coated capillary (50 μm I.D., 360 μm O.D.)^27,28^ was used for separation, and 100 nL of the sample was loaded into the capillary at 5 psi for 19 s. A glass emitter (orifice size: 30–35 μm) on the interface was filled with sheath liquid (0.2% FA and 10% methanol, pH 2.2) to produce electrospray at 2.2–2.5 kV. With 5% (v/v) acetic acid used as the background electrolyte (BGE), 30 kV voltage was applied to the injection end for 110 min, followed by 30 kV voltage and 10 psi pressure applied for 10 min to flush the capillary.

RPLC separation was performed on an EASY-nLC 1200 system (Thermo Fisher Scientific). Mobile phase A comprises 0.1% FA and 5% ACN in water, and mobile phase B is 0.1% FA in water/ACN (20:80). With the p-tau digest diluted 10 times with mobile phase A, 2 μL of the sample was loaded onto an RPLC capillary column (75 μm I.D., 50 cm length, C18 beads, 2 μm, 100 Å pore size; Thermo Fisher Scientific). RPLC gradient was processed from 2% B to 8% B in 5 min, from 8% B to 35% B in 75 min, and from 35% B to 80% B in 5 min and then stayed at 80% B for 15 min at a flow rate of 180 nL/min.

A Q Exactive HF (Thermo Fisher Scientific) mass spectrometer was used for CZE-MS/MS and RPLC-MS/MS analyses. The data were collected in data-dependent acquisition (DDA) mode. For a full MS scan, the resolution was set to 60,000 (at *m*/*z* of 200), the *m*/*z* range was 300–1500, the AGC target was 3e6, the maximum injection time was 50 ms, and the loop count was 10 (top 10). Fragmentation was performed with an isolation window of 2 *m*/*z*. Different normalized HCD energies, including 25, 28, and 30, were investigated. MS/MS data were acquired at a resolution of 60,000 (at *m*/*z* 200), *m*/*z* range of 200–2000, minimal AGC target of 1e4, and maximum injection time of 200 ms. The ion intensity threshold was set to 5e4, and the dynamic exclusion window was 30 s.

### cIEF-MS/MS-Based Top-Down Analyses of Intact p-tau Proteoforms

50 μg of purified p-tau protein (0N3R, in 0.1 mM NaCl and 20 mM Tris, pH 7.5) was loaded onto Amicon 10 kDa molecular weight cutoff centrifugal filters and centrifuged at 14,000 *g* for 15 min at 10 °C. The samples were washed four times with 10 mM NH_4_Ac. Eventually, 50 μL of the sample (∼1 mg/mL) was recovered from each filter. For cIEF separation, the samples were further incorporated with ampholytes and glycerol to form a mixture containing 0.5 mg/mL protein, 1.5% ampholyte (a narrow range of 8–10.5 and a wide range of 3–10 at a ratio of 4:1), and 15% glycerol.

The cIEF separation was performed on the same CE system for bottom-up analysis using a 1 m LPA-coated capillary. The sheath liquid was 0.2% FA and 10% methanol, unless otherwise stated. The capillary was filled with a 6 cm catholyte (0.5% NH_3_·H_2_O, 15% glycerol) at 10 psi for 11 s, followed by a 30 cm sample plug at 10 psi for 56 s. After sample injection, the capillary inlet was moved to an anolyte (0.1% FA, 15% glycerol) with a voltage of 20 kV applied for protein focusing and mobilization. At 20 min, an additional pressure of 0.2 psi was applied to assist protein mobilization for 120 min.

An Orbitrap Exploris 480 mass spectrometer (Thermo Fisher Scientific) was used for the cIEF-MS analysis with an in-house constructed CE-MS interface. The ion transfer tube temperature was set at 320 °C, the RF lens was set at 60%, and the intact protein mode was turned on in low-pressure mode. For DDA, a full MS scan was acquired at a low resolution of 7,500 (at *m*/*z* of 200), *m*/*z* range of 600–2500, normalized AGC target of 300%, and 10 microscans. The top 6 most intense precursors (charge states of 5–60 and a minimal intensity of 1e4) in full MS spectra were selected for fragmentation (an isolation window of 2 *m*/*z* and an HCD energy of 30%). MS/MS data were collected at a resolution of 60,000 (at *m*/*z* 200), *m*/*z* range of 200–2000, 10 microscans, normalized AGC target of 100%, and auto maximum injection time. The dynamic exclusion was applied with a duration of 30 s and with the exclusion of isotopes enabled.

### Data Analysis for Bottom-Up and Top-Down Proteomics

For the bottom-up experiment, the raw files for CZE-MS/MS and RPLC-MS/MS were analyzed by Proteome Discoverer (v 2.2). The raw files were searched against the sequence of human fetal-tau (Uniprot ID P10636-2) and *Escherichia coli* (*E. coli*, 4,593 entries, Aug 24, 2023) sequence database downloaded from UniProt (https://www.uniprot.org/). The precursor and fragment ion mass tolerances were 10 ppm and 0.02 Da, respectively. Trypsin was selected as the protease, with up to three missed cleavages. The dynamic modifications were oxidation (+15.995) on methionine (M), phosphorylation (+79.966) on serine (S)/threonine (T)/tyrosine (Y), acetylation (+42.011) on lysine (K)/arginine (R) and protein N-terminus, succinylation (+100.016) on lysine (K), and methylation (+14.016) on lysine (K)/arginine (R). Carbamidomethylation (+57.021) on cysteine (C) was set as a static modification. The target and decoy approach was used to determine the false discovery rates (FDRs) of the identifications. The FDRs were 1% for both the peptide and protein. For the modified peptides, only the PTM sites with a localization probability higher than 75 were included. The hydrophobicity of the peptides was obtained with the Peptide Analyzing Tool from ThermoFisher Scientific (https://www.thermofisher.com/us/en/home/life-science/protein-biology/peptides-proteins/custom-peptide-synthesis-services/peptide-analyzing-tool.html).

For the top-down experiment, charge state spectra were deconvoluted by the programs UniDec version 6.0.4^29^ and ESIprot (ESIprot Online (bioprocess.org)).^30^ For UniDec, the charge range is 1–100, while the mass range is 5000–1,000,000, with a sample mass recorded every 1 Da. The automatic *m*/*z* peak width was enabled. The MS2 spectra were processed using ProSight Lite software^31^ after deconvolution with TopPIC (top-down MS-based proteoform identification and characterization) software (version 1.6.3).^32^

### Experimental and Predicted Electrophoretic Mobility (μ_ef_)

The prediction was performed according to the published literature.^33^ The electro-osmotic flow was assumed zero in the LPA-coated capillary with 5% AA (pH 2.4) as the BGE. For calculating the experimental μ_ef_, eq 1 was used:
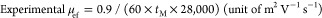
1where migration times (*t*_M_, min) were determined as the time obtained from the database search results. To calculate the predicted μ_ef_, eq 2 was used:
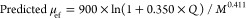
2where *Q* is the number of charges of each peptide in the liquid phase, represented by the number of positively charged amino acid residues in the peptide sequence (K, R, H, and N-terminus), and *M* is the molecular mass equal to the mass reported by the search engine in Da.

## Results and Discussion

### CZE-MS/MS Is an Efficient Analytical Method for Bottom-Up Proteomics of Human p-tau Protein

Due to the high separation efficiency and high detection sensitivity, CZE-MS is a very efficient analytical method for the analysis of peptides and proteins.^19^ In this work, we employed both CZE-MS/MS and RPLC-MS/MS for the analysis of tryptic peptides of the shortest recombinant human tau isoform, 0N3R, phosphorylated by cyclin-dependent kinase 5 (CDK5).^34,35^ We used *p-tau-0N3R* to represent the CDK5-treated tau-0N3R protein throughout the manuscript. Apart from the most common tau PTM, phosphorylation, many other ways, such as glycosylation, acetylation, glycation, ubiquitylation, *O*-GlcNAcylation, aggregation, and filament formation, can be modified on tau protein in neurodegenerative disease brain.^36^ While the current p-tau was synthesized in *E. coli* for the creation of a human AD-relevant hyperphosphorylation pattern,^8,23^ additional PTMs, such as methylation,^37^ acetylation,^38^ and succinylation^39,40^ that are known to occur in many recombinant proteins produced in *E. coli*, were also present in this and other PIMAX p-tau preparations (see below and unpublished observations by Hagar and Kuo).

RPLC-MS/MS identified peptides covering 96% of the *p-tau-0N3R* sequence (Figure S1A), while CZE-MS/MS achieved 100% sequence coverage (Figure S1B). As shown in Supporting Information II, CZE-MS/MS identified more tau peptides than RPLC-MS/MS (205 vs 159), even with a two times lower sample consumption. Compared with RPLC-MS/MS, CZE-MS/MS could detect peptides with lower abundance, evidenced by the much wider intensity dynamic range of identified peptides (5 vs 4 orders of magnitude; Figure S1C). The data indicate a high sensitivity of CZE-MS/MS for measuring tau peptides.

**Figure 1:**
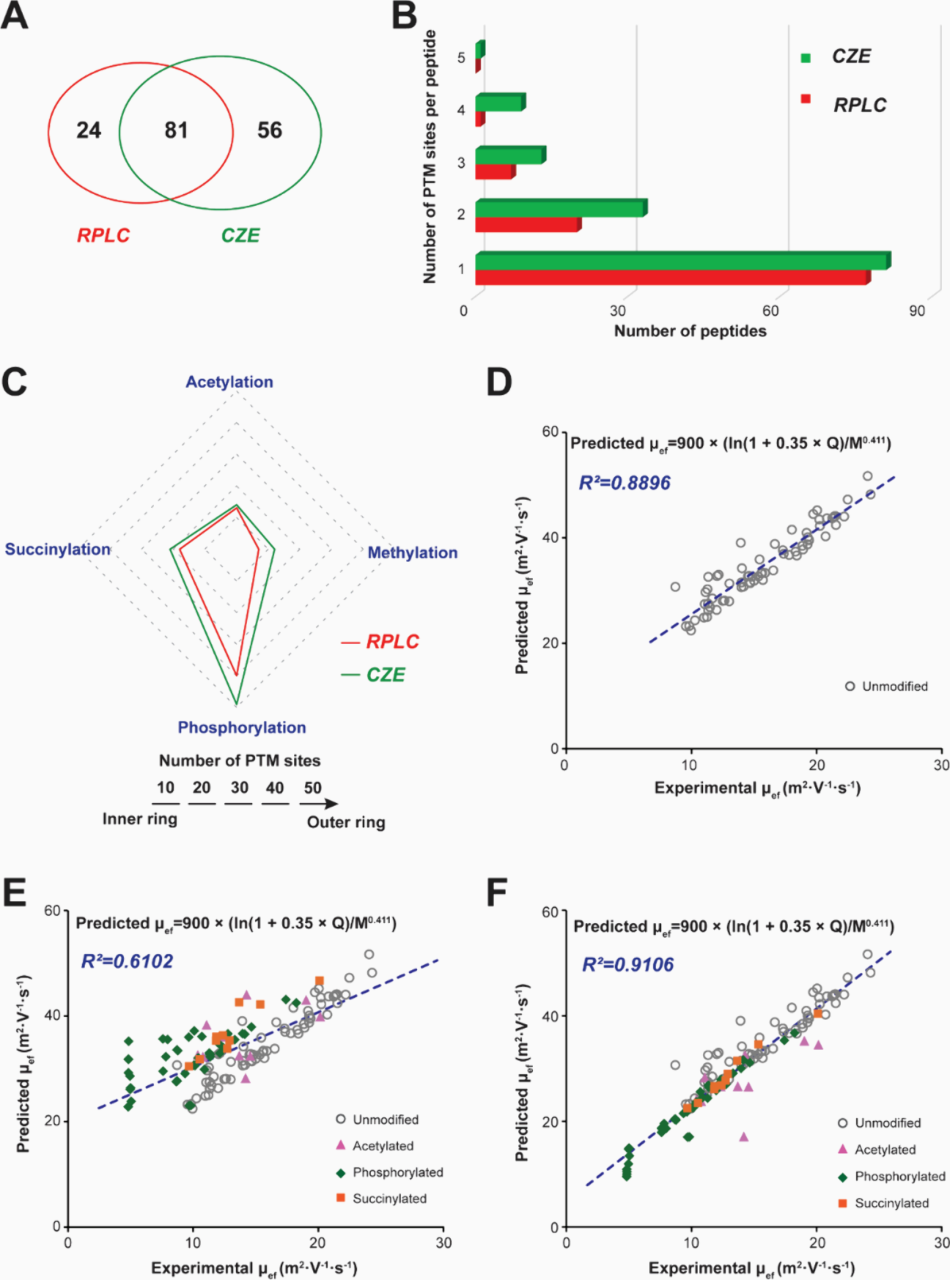
CZE-MS/MS and RPLC-MS/MS analyses of tryptic digest from the full-length human phosphorylated tau-0N3R (*p-tau-0N3R*) protein. Comparison of the (A) number of peptides with PTM, (B) number of PTM sites per peptide, and (C) total number of PTM sites identified by RPLC-MS/MS and CZE-MS/MS. Linear correlations between theoretical and experimental μ_ef_ of (D) unmodified peptides and (E) unmodified plus phosphorylated, acetylated, and succinylated peptides identified from the p-tau sample by CZE-MS/MS analysis under the BGE of 5% (v/v) acetic acid (pH 2.4). (F) Linear correlations between theoretical and experimental μ_ef_ values of unmodified peptides plus phosphorylated, acetylated, and succinylated peptides after charge Q corrections due to PTMs. In panel (F), we adjusted the Q by −1, −1, and −1 for peptides with phosphorylation, acetylation, and succinylation, respectively. The gray circles represent peptides without PTMs, the purple triangles represent acetylated peptides, the green diamonds represent phosphorylated peptides, and the orange squares represent succinylated peptides.

Among the identified tau peptides, 77% of the peptides carrying PTM sites were commonly identified by RPLC-MS/MS and CZE-MS/MS, showing the different peptide identification preferences for these two methods, as shown in Figure 1A. As shown in Figure 1B, both methods can identify peptides with multiple PTM sites, with up to four occurrences per peptide. In addition, CZE-MS/MS could identify more peptides carrying the phosphate group or succinyl group (Figure 1C). RPLC separates peptides based on their hydrophobicity, whereas CZE enables peptide separation according to their electrophoretic mobilities, corresponding to their charge-to-size ratios. As shown in Figure S1D, RPLC-MS/MS tends to identify more hydrophobic peptides than CZE-MS/MS because the retention of very hydrophilic peptides on RPLC is an issue. When peptides are phosphorylated, they become more hydrophilic due to the introduction of more charges. The charge changes of peptides due to phosphorylation could result in substantial changes in the charge-to-size ratio, leading to better separations by CZE than by RPLC. Compared to RPLC-MS/MS, the tau peptides with one/multiple phosphorylation sites can be better identified by CZE-MS/MS, Figure S1E. Our results demonstrate the high complementarity of these two methods for tau peptide identifications and tau PTM analysis.

We applied a multiparametric sequence-specific model^41^ to predict the electrophoretic mobility for the peptides identified by CZE-MS/MS, which could help validate the confidence of peptide identifications by examining the correlation between predicted and experimental μ_ef_ of peptides. In the Experimental Section, we described the details of calculating the predicted and experimental μ_ef_ values of the peptides.

First, the peptides without any PTMs produced a linear correlation coefficient (*R*^2^) of 0.8896 (Figure 1D). Then, the μ_ef_ prediction equation was used for p-tau peptides with PTMs (acetylation, phosphorylation, and succinylation). Since those PTMs usually lead to reduced mobility of peptides, most of the p-tau peptides with PTMs were clearly off the trendline without charge Q corrections (Figure 1E). Peptide acetylation and phosphorylation were reported to reduce the charge Q by roughly one unit, according to our previous studies.^41^ According to the chemical reaction involved in succinylation, we expect that one succinylation reduces the peptide charge Q by one unit under our pH 2.4 BGE condition. After we applied charge Q reduction for those acetylated, phosphorylated, and succinylated peptides, the linear correlation coefficient between predicted and experimental μ_ef_ increased from 0.6102 (Figure 1E) to 0.9106 (Figure 1F). The data demonstrate that the μ_ef_ of modified tau peptides can be predicted accurately and that one succinylation PTM certainly reduced the peptide charge Q by one unit under our CZE-MS condition. An example of the peptide carrying 1, 2, or 3 phosphate groups identified by CZE-MS/MS is shown in Figure S2. Peptides that carry more phosphate groups migrate more slowly. As illustrated in Figure S3, in comparison with the unmodified peptide, the peptides with the same sequence but carrying one phosphate group or succinyl group were eluted later at almost the same time, which agrees well with the prediction result.

**Figure 2:**
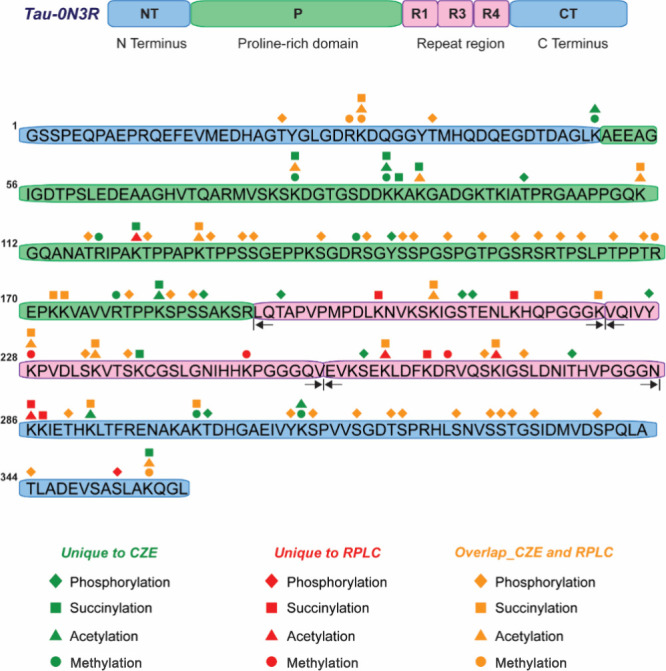
Bar diagram of human *p-tau-0N3R* with the distribution of observed PTM sites. The diamond, square, triangle, and circle represent the possible phosphorylation, succinylation, acetylation, and methylation sites, respectively, identified from bottom-up proteomics. The green and red symbols represent the PTM sites uniquely identified by CZE-MS/MS and RPLC-MS/MS, respectively. The orange symbols represent the PTM sites commonly identified by CZE-MS/MS and RPLC-MS/MS.

We combined all of the PTM sites identified by RPLC-MS/MS and CZE-MS/MS (Figure2). Interestingly, it was found that more PTM sites located in the proline-rich domain were discovered by CZE-MS/MS, while RPLC-MS/MS favored the identification of PTM sites of the C-terminal half, further demonstrating the high complementarity between these two methods for PTM identification of tau. The PTM mapping showed that succinylation occurred widely along the tau sequence, and lysines that could be methylated or acetylated are also frequent targets of succinylation, which agrees well with the literature.^42^

Among the 67 putative phosphorylation sites on *p-tau-0N3R*, the number of P-sites identified on serine (S), threonine (T), and tyrosine (Y) residues on tau was 40 sites (26 S, 13 T, and 1 Y) from RPLC-MS/MS and 49 sites (27 S, 19 T, and 3 Y) from CZE-MS/MS (Table S1). The total number of 50 detected phosphorylated residues represents 50/67 = 75% of all potential phosphorylated residues, which occupied almost all of the potential phosphorylation sites in the repeat region (10/13) and C-terminal domains (15/17).

Hanger et al. compiled the phosphorylation sites for tau protein, with 12 phosphorylation sites detected from the CDK5 phosphorylated tau protein.^43^ Kuo and colleagues applied four endoproteinases, trypsin, Lys-C, Arg-C, or Asp-N, for in-gel digestion, followed by RPLC-MS/MS, and identified 31 phosphorylation sites from human tau-0N3R.^9^ As shown in Table S2, all phosphorylation sites observed by Hanger et al. were detected in our work. In comparison with Kuo’s result, most of the phosphorylation sites, except Thr-53, Thr-91, and Ser-235, were identified by our method. Among the 21 phosphorylation sites uniquely identified in our result, 7 of them were uniquely identified by the CZE-MS/MS method, which further indicated the high promise of CZE-MS/MS for a global characterization of tau PTMs.

### cIEF-MS Analysis of Intact Human p-tau-0N3R Proteoforms

Our bottom-up proteomics measurement reveals rich PTM information (e.g., 50 phosphorylation sites) on human *p-tau-0N3R*. However, we cannot figure out the pictures of intact *p-tau-0N3R* proteoforms based on the bottom-up proteomics data. For example, what types of *p-tau-0N3R* proteoforms are there? How are different PTMs organized on each *p-tau-0N3R* proteoform? Top-down proteomics measurement of tau proteins is critical for filling this knowledge gap and is fundamental for understanding the roles of tau protein in AD progression because tau functions as intact proteoforms carrying various combinations of PTMs, not individual peptides carrying PTMs. Therefore, in this work, we also investigated CE-MS for top-down proteomics analysis of human *p-tau-0N3R*.

Top-down proteomics has been tested for characterizing intact tau proteoforms in the literature.^10,16^ Mandelkow et al. analyzed the intact phosphorylated tau (2N4R) expressed in eukaryotic cells by direct infusion native MS, revealing the distribution of the number of phosphates per tau protein.^13^ Loo et al. integrated ion mobility spectrometry and electron capture dissociation by direct infusion native MS for the intact tau and tau/CLR01 complex to pinpoint the site(s) of inhibitor binding.^46^ Online liquid-phase high-resolution tau proteoform separation prior to MS is essential for the detection of low-abundance tau proteoforms and the generation of a complete tau proteoform landscape.

**Figure 3:**
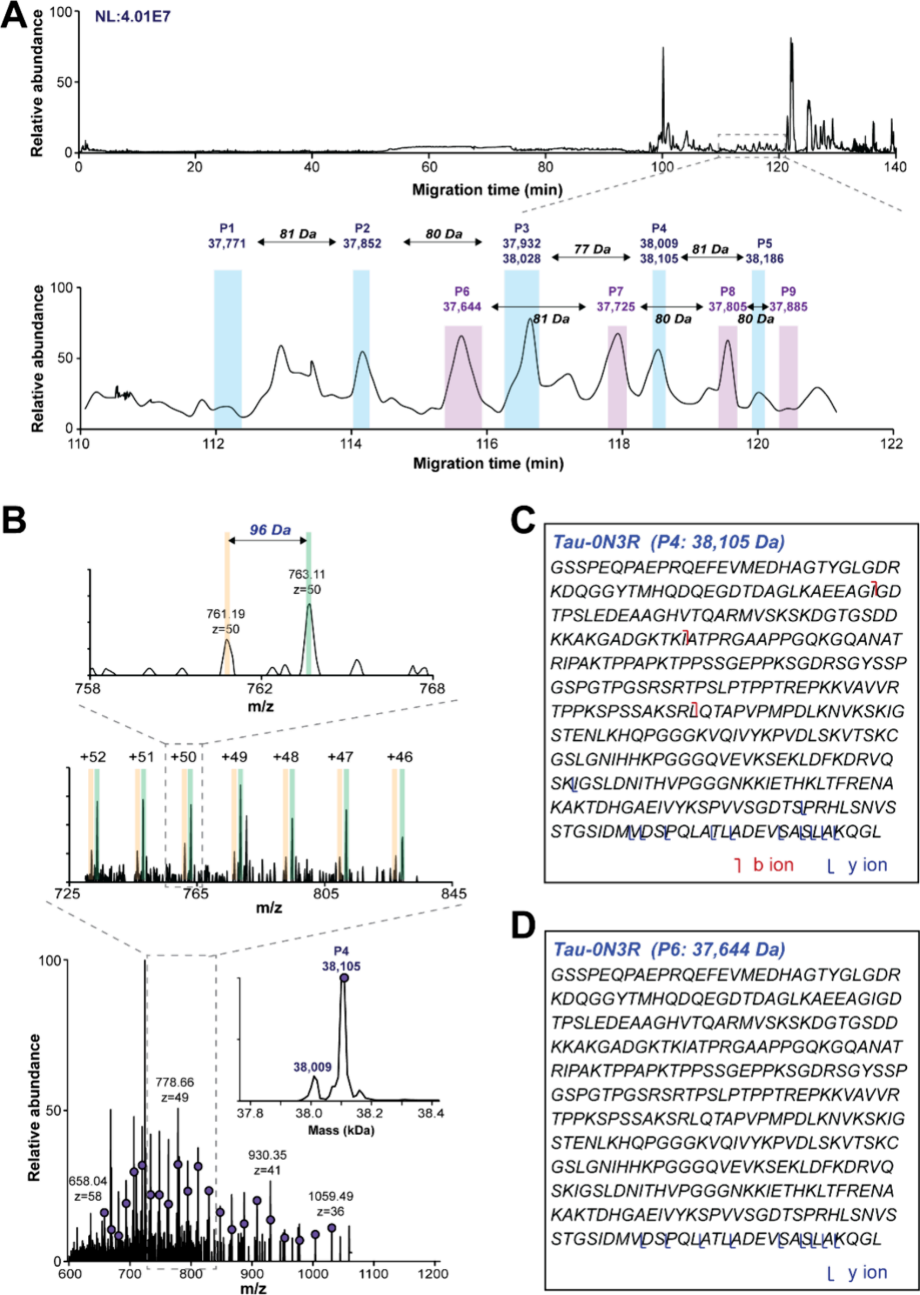
cIEF-MS analysis of human *p-tau-0N3R* under denaturing conditions. (A) Base peak electropherogram of *p-tau-0N3R* proteoforms (top). Zoomed-in base peak electropherogram in 110–122 min with deconvoluted masses of *p-tau-0N3R* proteoforms labeled. (B) Averaged mass spectra and deconvoluted mass spectra (inserted figure) of *p-tau-0N3R* proteoforms detected in peak 4 (P4). A zoomed-in mass spectrum of *p-tau-0N3R* proteoforms was detected in P4. The sequence and fragmentation pattern of *p-tau-0N3R* proteoforms detected are shown in (C) P4 and (D) P6.

In this work, we first tested CZE-MS to characterize the *p-tau-0N3R* proteoforms. However, we did not observe clear intact tau proteoform signals, maybe due to the high complexity of the *p-Tau-0N3R* proteoforms and coelution with other background proteins. We decided to investigate cIEF-MS for this purpose due to the super high separation resolution of cIEF for proteins/proteoforms with isoelectric point (pI) difference and its much higher sample loading capacity, which could be critical for highly complex and large proteoforms. We performed cIEF-MS analysis of *p-tau-0N3R* with about 300 ng of tau protein injected and detected 11 potential *p-tau-0N3R* proteoforms after mass deconvolution with UniDec software within 9 electrophoretic peaks (Figure 3A). We further confirmed the deconvolution results from UniDec using ESIprot software.^30^ The standard deviation of the deconvoluted proteoform mass was less than 2.5 Da. The mass of detected potential 0N3R proteoforms is in the range of 37.6 and 38.2 kDa. The average mass of tau-0N3R proteoforms, with N-terminal methionine removal and a seven amino acid residue remnant at the N-terminus from cloning (GSSPEQP), and without any PTMs, is 37 312 Da. Therefore, the proteoform in P1 (37 771 Da) might represent a tau-0N3R proteoform containing 5 phosphate groups, 1 acetyl group, and 1 methyl group. The proteoforms in P2 (37 852 Da) and P3 (37 932 Da) show an around 80 Da mass difference with P1, which might carry 6 and 7 phosphate groups, respectively. The original mass spectra and deconvoluted masses are shown in Figure S4A–C.

We detected another proteoform with 38,028 Da in P3, with a + 96 Da mass shift in comparison with the proteoform of 37,932 Da (Figure S4C). We also observed two proteoforms in peak 4 with masses of 38,009 and 38,105 Da (Figure 3B). Interestingly, the mass difference between 37,932 Da in peak 3 and 38,009 Da in peak 4 is close to 80 Da, and a similar mass difference was also observed for the other two proteoforms in peaks 3 and 4 (i.e., 38,028 vs 38,105 Da). Peak 5 contains one proteoform (38,186 Da; Figure S4D) close to the mass of 0N3R, which is 81 Da heavier than one proteoform in peak 4 (38,105 Da). The data suggest that the tau-0N3R proteoforms in peaks 4 and 5 should carry 8 and 9 phosphate groups. An observation from the data is that the *p-tau-0N3R* proteoform carrying more phosphate groups migrated more slowly due to the reduction of the isoelectric point (pI). Also, the migration time reduction by adding one phosphate group is substantial (i.e., about 2 min) (Figure 3A). Based on the results, we speculate that the 96 Da mass shift between the two proteoforms in peak 3 or peak 4 should not correspond to a phosphorylation PTM because they nearly comigrated. We also detected peak series corresponding to another four *p-tau-0N3R* proteoforms (peaks 6–9), with masses ranging from 37,644 to 37,885 Da, equally spaced by ∼80 Da (Figures 3A and S4E–H). The proteoform in P6 (37,644 Da) might be a tau-0N3R proteoform carrying 4 phosphate groups and 1 methyl group, while those proteoforms from P7 to P9 also might be from *p-tau-0N3R* with an increased amount of phosphorylation. We further analyzed the MS/MS data of peaks 4 and 6 and did the targeted analysis using ProSight Lite^31^ (Figure 3C,D). A series of fragment ions of these two peaks matched the C-terminus of tau-0N3R, which agrees with the manual annotation data (Figure S5). We also observed several fragment ions corresponding to the neutral loss of phosphoric acid during manual annotation (Figure S5), confirming that those proteoforms are from phosphorylated tau-0N3R. However, we cannot localize the PTM sites on the tau proteoforms due to the high positional heterogeneity of the phosphorylation modifications and the limited fragmentation efficiency of higher energy collision dissociation (HCD) on those large proteoforms. Electron-based fragmentation techniques [e.g., electron-capture dissociation (ECD) and electron-transfer dissociation (ETD)] have shown much more extensive backbone cleavage than HCD for large proteoforms and can maintain labile PTMs (e.g., phosphorylation) on fragments for more accurate localization.^44−48^ For example, Nshanian et al. applied ECD to characterize fragments of 4R tau, with the production of rich fragment ions enabling the localization of phosphorylation sites and even the determination of inhibitor binding sites.^46^ In addition, ultraviolet photodissociation (UVPD) has shown impressive backbone cleavage coverages of large proteoforms,^49,50^ and it could be a valuable technique to delineate tau proteoforms. Overall, the results demonstrate that cIEF-MS is a promising technique for characterizing tau proteoforms with only nanograms of sample consumption. In future work, we will try to combine cIEF-MS with ECD/ETD or UVPD fragmentation methods to pinpoint the phosphorylation sites of each Tau proteoform.

### cIEF-MS Analysis of Intact Human p-tau-0N3R Protein under Pseudonative Conditions

**Figure 4:**
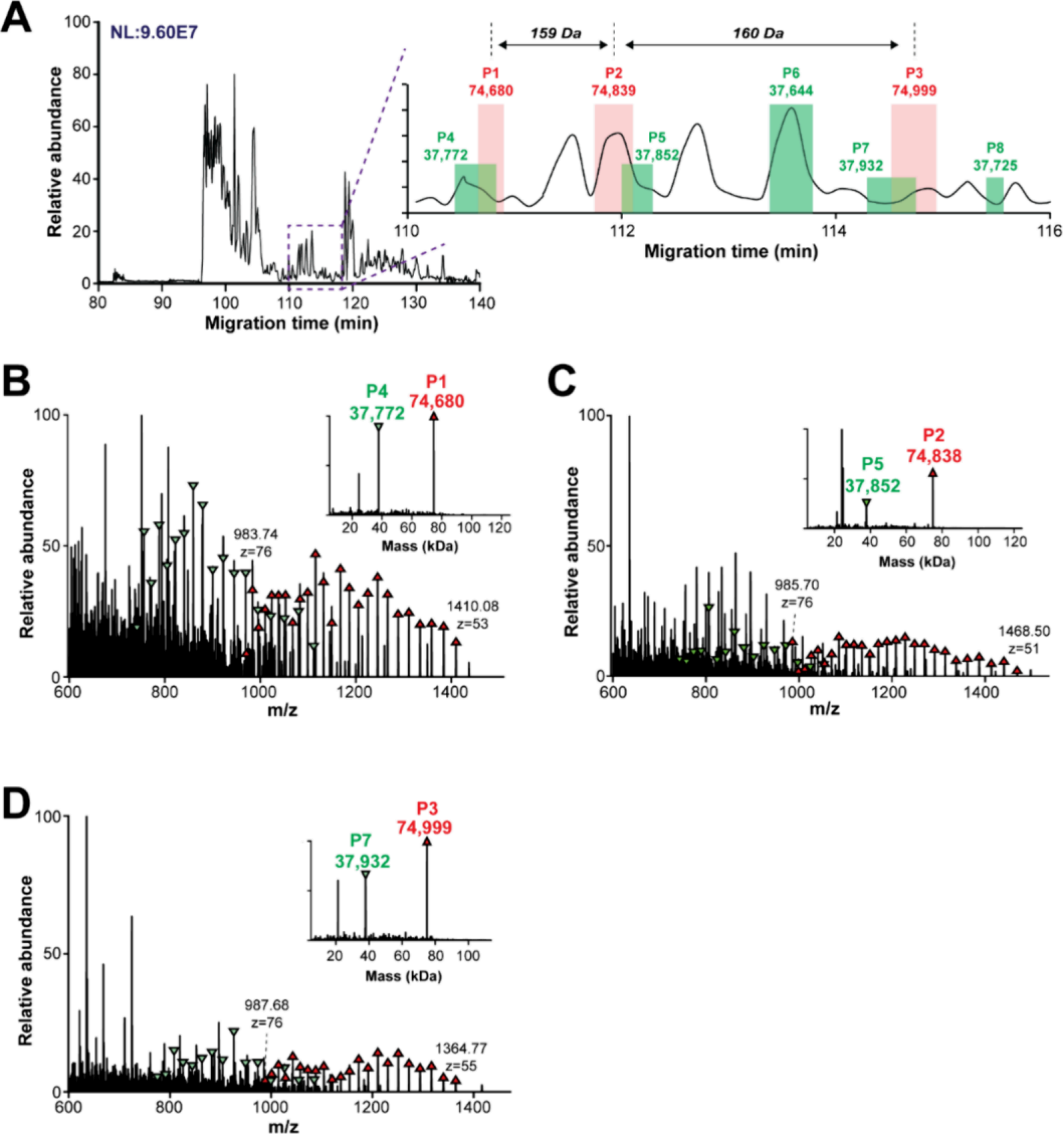
cIEF-MS analysis of human *p-tau-0N3R* under pseudonative conditions. (A) Base peak electropherogram of *p-tau-0N3R* proteoforms with a zoomed-in base peak electropherogram of the 110–116 min region (inserted figure). The deconvoluted masses of different tau proteoforms are labeled. (B–D) Averaged mass spectra and deconvoluted masses (insets) of *p-tau-0N3R* proteoforms detected in P1–P3.

We also performed a top-down MS analysis of *p-tau-0N3R* under pseudonative conditions by changing the sheath liquid from 10% methanol and 0.2% FA to 10 mM ammonium acetate (pH adjusted to 4 with acetic acid). We have demonstrated native cIEF-MS for the characterization of protein complexes and antibody–drug conjugates in one of our recent studies.^51^ Here, we applied a similar condition to that for *p-tau-0N3R*, aiming to achieve more information about the tau protein sample. As expected, with the increase in pH of the sheath liquid, the most abundant charge state of the detected *p-tau-0N3R* proteoforms shifted from around +50 (Figure S4) to around +40 (Figure S6). We detected some of the same proteoforms under denaturing conditions. For example, the proteoforms in P4, P5, P6, P7, and P8 in Figure 4A are the same as those in P1, P2, P6, P3, and P7 in Figure 3A.

Interestingly, the pseudonative condition also resulted in the detection of three large proteoforms, ranging from 74,680 to 74 999 Da, equally spaced by ∼160 Da (Figure 4A). We speculate that those over 74 kDa are dimers of different *p-tau-0N3R* proteoforms. Considering the partial comigration of proteoforms in P1 and P4, P2 and P5, and P3 and P7, a possible explanation is that the proteoforms in P4, P5, and P7 might contribute to the formation of dimers in P1, P2, and P3. For example, the proteoform dimer of 74 680 Da detected in P1 might be formed by the two tau-0N3R proteoforms of 37,772 (tau-0N3R proteoform carrying 5 phosphate groups), with an unknown −864 Da mass shift. Similarly, the large proteoforms detected in P2 and P3 are the dimers carrying 6 and 7 phosphate groups, respectively. These results are in agreement with that the pathological tau protein could form dimers.^52,53^ Although the assumption needs to be confirmed with a more efficient fragmentation method for better tau proteoform characterization, it demonstrates the high promise of pseudonative cIEF-MS/MS for the top-down MS analysis of specific tau proteoforms in their aggregated forms. Importantly, our preliminary data suggest that the dimer formation of *p-tau-0N3R* proteoforms may be selective because proteoforms in P4, P5, and P7 are involved, while P6 and P8 are not involved.

## Conclusions

In this pilot study, for the first time, CE-MS/MS was applied for bottom-up and top-down proteomics analyses of human *p-tau-0N3R* expressed in *E. coli* cells. CZE-MS/MS-based bottom-up proteomics analysis mapped 49 phosphorylation sites, 14 acetylation sites, 12 methylation sites, and 21 succinylation sites. CZE-MS/MS provided more PTM sites of *p-tau-0N3R* than RPLC-MS/MS, and the two techniques showed nice complementarity regarding tau PTM characterization. CIEF-MS/MS-based top-down analysis of *p-tau-0N3R* proteoforms offered a bird’s-eye view of tau proteoforms in the sample with high sensitivity. The pseudonative cIEF-MS revealed the likely formation of 0N3R dimers from specific proteoforms. The results show that CZE-MS/MS and cIEF-MS/MS are very useful analytical tools for elucidating tau PTMs and their proteoforms.

## Data Availability

MS raw data and result files have been deposited to the ProteomeXchange Consortium via the PRIDE repository^54^ and are publicly accessible from its website with the data set identifier PXD053367.

## Supporting Information Available

Phosphorylation sites of the human *p-tau-0N3R* protein identified by RPLC-MS/MS and CZE-MS/MS with localization probability higher than 75 (Table S1); P-sites in human tau (0N3R) expressed in *E. coli* cells by three independent studies (Table S2); bottom-up analysis of the phosphorylated human *p-tau-0N3R* protein via RPLC-MS and CZE-MS/MS (Figure S1); example spectra of peptides carrying different numbers of phosphate groups (Figure S2); example spectrum of peptide (KDQGGYTMHQDQEGDTDAGLK) without PTMs and with phosphorylation or succinylation (Figure S3); cIEF-MS analysis of human *p-tau-0N3R* under denaturing conditions (Figure S4); annotated MS/MS spectra of two human *p-tau-0N3R* proteoforms carrying different numbers of phosphate groups under denaturing conditions (Figure S5); and averaged mass spectra and deconvoluted masses of *p-tau* proteoforms under pseudonative conditions (Figure S6) (PDF)
Lists of tryptic peptides identified from human *p-tau-0N3R* by RPLC-MS/MS and CZE-MS/MS (XLSX)
