# Supplementary material for: Pilot Study for Deciphering Post-Translational Modifications and Proteoforms of Tau Protein by Capillary Electrophoresis-Mass Spectrometry
Source: J Proteome Res. 2024 Sep 27;23(11):5085–95. doi: 10.1021/acs.jproteome.4c00587 (PMC11536466; doi:10.1021/acs.jproteome.4c00587)
Supplement: Supplementary file 1 — pr4c00587_si_001.pdf [file pr4c00587_si_001.pdf]

Supporting Information I

A pilot study for deciphering post-translational modifications and proteoforms of tau protein by capillary electrophoresis-mass spectrometry

Fei Fang[1#], Tian Xu[1#], Hsiao-Tien Chien Hagar[2], Stacy Hovde[2], Min-Hao Kuo[2*], Liangliang Sun[1*]

[1] Department of Chemistry, Michigan State University, 578 S Shaw Lane, East Lansing, MI 48824, USA

[2] Department of Biochemistry and Molecular Biology, Michigan State University, 603 Wilson Road, Room 401, East Lansing, MI, 48824, USA

[#] Those two authors contributed equally to this work.

[*] Corresponding authors.

Email: lsun@chemistry.msu.edu

Phone: 517-353-0498

kuom@msu.edu

Phone: 517-355-0163

Table of Contents

Supplementary Tables S1-S2 and Figures S1-S6 (pdf), Supporting Information II (excel).

**Table S1**	The phosphorylation sites of human *p-tau-0N3R* protein identified by RPLC-MS/MS and CZE-MS/MS with localization probability higher than 75
**Table S2**	P-sites in human tau (0N3R) expressed in E. coli cells by three independent studies
**Figure S1**	Bottom-up analysis of phosphorylated human *p-tau-0N3R* protein via RPLC-MS and CZE-MS/MS
**Figure S2**	Example spectra of peptides carrying different numbers of phosphate groups
**Figure S3**	Example spectrum of peptide (KDQGGYTMHQDQEGDTDAGLK) without PTMs and with phosphorylation or succinylation
**Figure S4**	cIEF-MS analysis of human *p-tau-0N3R* under denaturing condition
**Figure S5**	Annotated MS/MS spectra of two human *p-tau-0N3R* proteoforms carrying different numbers of phosphate groups under denaturing conditions
**Figure S6**	Averaged mass spectra and deconvoluted masses of *p-tau* proteoforms under pseudo-native condition
**Supporting Information II (XLSX)**	Lists of tryptic peptides identified from human *p-tau-0N3R* by RPLC-MS/MS and CZE-MS/MS

Table S1. The phosphorylation sites of *p-tau-0N3R* protein identified by RPLC-MS/MS and CZE-MS/MS with localization probability higher than 75.

Peptide No.	Residues	P-Site	Peptide sequence	RPLC	CZE
1	6-23	T17	QEFEVMEDHAGTYGLGDR	+	+
2	25-44	T30	DQGGYTMHQDQEGDTDAGLK	+	+
3	91-97	T95	TKIATPR		+
4	106-116	T111	GQANATRIPAK	+	+
5	113-122	T117	IPAKTPPAPK	+	+
6	123-132	T123	TPPSSGEPPK	+	+
7	123-132	S126	TPPSSGEPPK	+	+
8	123-151	S127	TPPSSGEPPKSGDRSGYSSPGSPGTPGSR	+	+
9	123-151	S133	TPPSSGEPPKSGDRSGYSSPGSPGTPGSR	+	+
10	123-151	S137	TPPSSGEPPKSGDRSGYSSPGSPGTPGSR	+	+
11	133-151	Y139	SGDRSGYSSPGSPGTPGSR		+
12	137-151	S140	SGYSSPGSPGTPGSR	+	+
13	123-151	S141	TPPSSGEPPKSGDRSGYSSPGSPGTPGSR	+	+
14	123-151	S144	TPPSSGEPPKSGDRSGYSSPGSPGTPGSR	+	+
15	137-151	T147	SGYSSPGSPGTPGSR	+	+
16	137-151	S150	SGYSSPGSPGTPGSR	+	+
17	152-166	S152	SRTPSLPTPPTREPK	+	+
18	152-163	T154	SRTPSLPTPPTR	+	+
19	154-163	S156	TPSLPTPPTR	+	+
20	154-163	T159	TPSLPTPPTR	+	+
21	154-166	T162	TPSLPTPPTREPK	+	+
22	167-182	T173	KVAVVRTPPKSPSSAK	+	+
23	173-182	S177	TPPKSPSSA	+	+
24	173-182	S179	TPPKSPSSAK	+	+
25	177-182	S180	SPSSAK		+
26	185-196	T187	LQTAPVPMPDLK		+
27	202-209	S204	IGSTENLK		+
28	202-209	T205	IGSTENLK		+
29	217-251	Y221	VQIVYKPVDLSKVTSKCGSLGNIHHKPGGGQVEVK		+
30	217-232	S227	VQIVYKPVDLSKVTSK	+	+
31	217-232	S231	VQIVYKPVDLSKVTSK	+	+
32	252-260	S252	SEKLDFKDR		+
33	261-280	S263	VQSKIGSLDNITHVPGGGNK	+	+
34	261-280	S267	VQSKIGSLDNITHVPGGGNK	+	+
35	265-280	T272	IGSLDNITHVPGGGNK		+
36	281-290	T284	KIETHKLTFR	+	+
37	295-317	T297	AKTDHGAEIVYKSPVVSGDTSPR		+
38	295-317	Y305	AKTDHGAEIVYKSPVVSGDTSPR	+	+
39	295-317	S307	AKTDHGAEIVYKSPVVSGDTSPR	+	+
40	307-317	S311	SPVVSGDTSPR	+	+
41	307-317	T314	SPVVSGDTSPR	+	+
42	307-317	S315	SPVVSGDTSPR	+	+
43	318-349	S320	HLSNVSSTGSIDMVDSPQLATLADEVSASLAK	+	+

44	318-349	S323	HLSNV**S**STGSIDMVDSPQLATLADEVSASLAK	+	+
45	318-349	S324	HLSNVS**S**TGSIDMVDSPQLATLADEVSASLAK	+	+
46	318-349	T325	HLSNVSS**T**GSIDMVDSPQLATLADEVSASLAK	+	+
47	307-349	S327	SPVVSGDTSPRHLSNVSSTG**S**IDMVDSPQLATLADEVSASLAK	+	+
48	318-349	S333	HLSNVSSTGSIDMVD**S**PQLATLADEVSASLAK	+	+
49	318-349	T338	HLSNVSSTGSIDMVDSPQLA**T**LADEVSASLAK	+	+
50	318-349	S346	HLSNVSSTGSIDMVDSPQLATLADEVSA**S**LAK	+	

Table S2. P-sites in human tau (0N3R) expressed in *E. coli* cells by three independent studies: Hanger et al. (2020) [compile], Kuo et al. (2020) [RPLC-MS/MS] and current work [CZE-MS/MS and RPLC-MS/MS]. Column 1, potential P-sites in full-length human tau (0N3R, residues S, T, Y); column 2, P-sites in human tau compiled by Hanger, 2020; columns 3, P-sites in tau observed in Kuo, 2020; column 4, P-sites in tau observed in current work. Additional sites observed in the current work are marked in red, and sites observed in previous but not in the present study are in blue.

Potential P-sites in hTau 0N3R	P-sites obs. (Hanger, 2020)	P-sites obs. (Kuo 2020)	P-sites obs. (this work)
T17			T17
Y18			
Y29			
T30			T30
T39			
T53		T53	
S55			
T65			
S71			
S73			
T77			
S79			
T91		T91	
T95	T95	T95	T95
T111		T111	T111
T117		T117	T117
T123	T123	T123	T123
S126			S126
S127			S127
S133		S133	S133
S137	S137	S137	S137
Y139			Y139
S140			S140
S141	S141	S141	S141
S144	S144	S144	S144
T147	T147	T147	T147
S150			S150
S152			S152
T154	T154	T154	T154
S156	S156		S156
T159		T159	T159
T162		T162	T162
T173	T173	T173	T173

S177	S177	S177	S177
S179			S179
S180			S180
S183			
T187		T187	T187
S200			
S204			S204
T205			S205
Y221			Y221
S227		S227	S227
T230			
S231		S231	S231
S235		S235	
S252			S252
S263		S263	S263
S267		S267	S267
T272		T272	T272
T284		T284	T284
T288			
T297			T297
Y305			Y305
S307	S307	S307	S307
S311		S311	S311
T314		T314	T314
S315	S315	S315	S315
S320		S320	S320
S323			S323
S324			S324
T325			T325
S327		S327	S327
S333		S333	S333
T338			T338
S344			
S346			S346

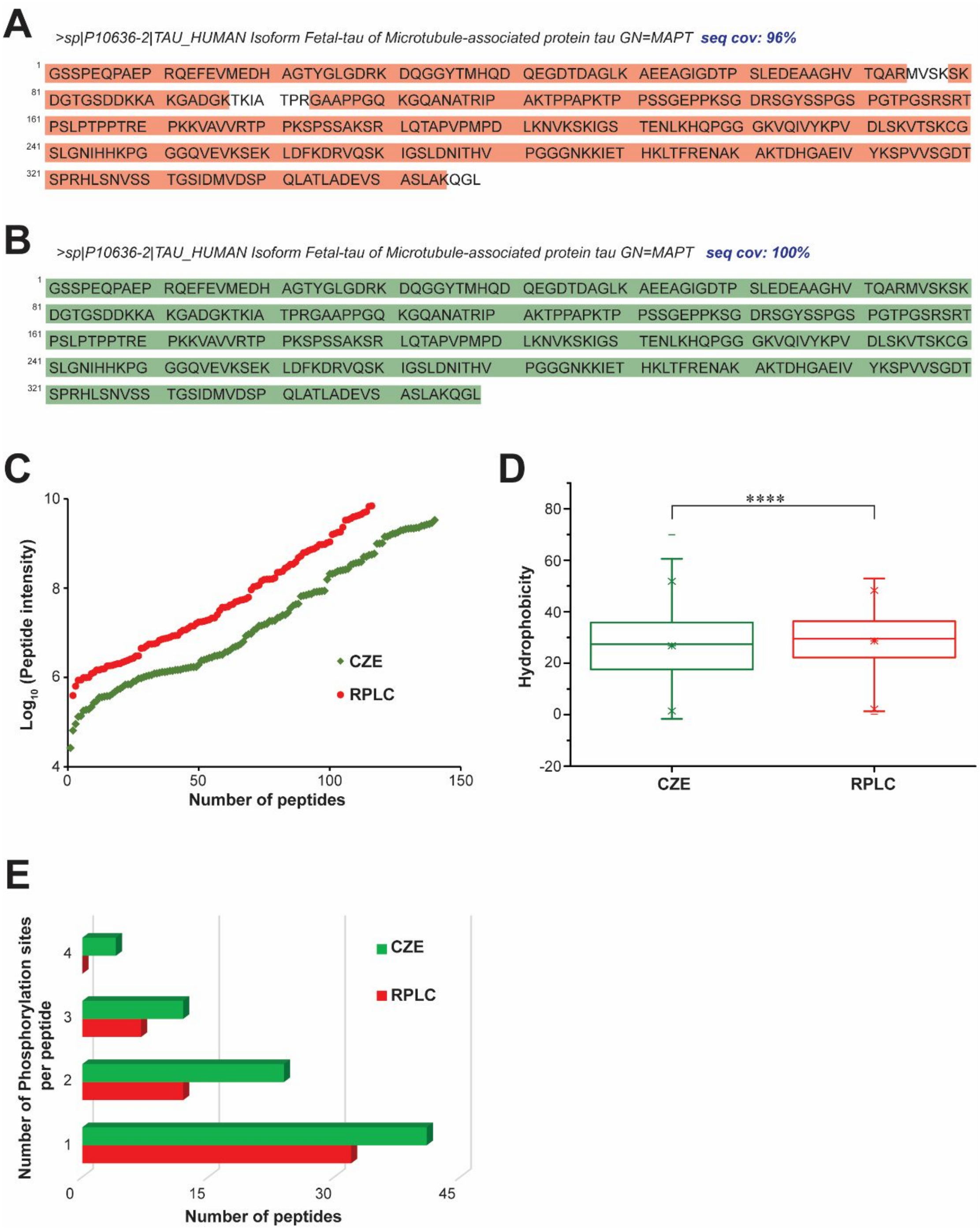

Figure S1. Bottom-up analysis of phosphorylated human *p-tau-0N3R* protein via RPLC-MS/MS and CZE-MS/MS. Coverage map of 0N3R isoform of human tau identified by (A) RPLC-MS/MS and (B) CZE-MS/MS. (C) Comparison of intensity of tau peptides identified from RPLC-MS/MS and CZE-MS/MS. (D) Hydrophobicity distribution of peptides identified from RPLC-MS/MS and CZE-MS/MS.****p < 0.0001, two-tailed, unpaired Mann-Whitney test. (E) Comparison of the number of phosphorylation sites per peptide identified from RPLC-MS/MS and CZE-MS/MS.

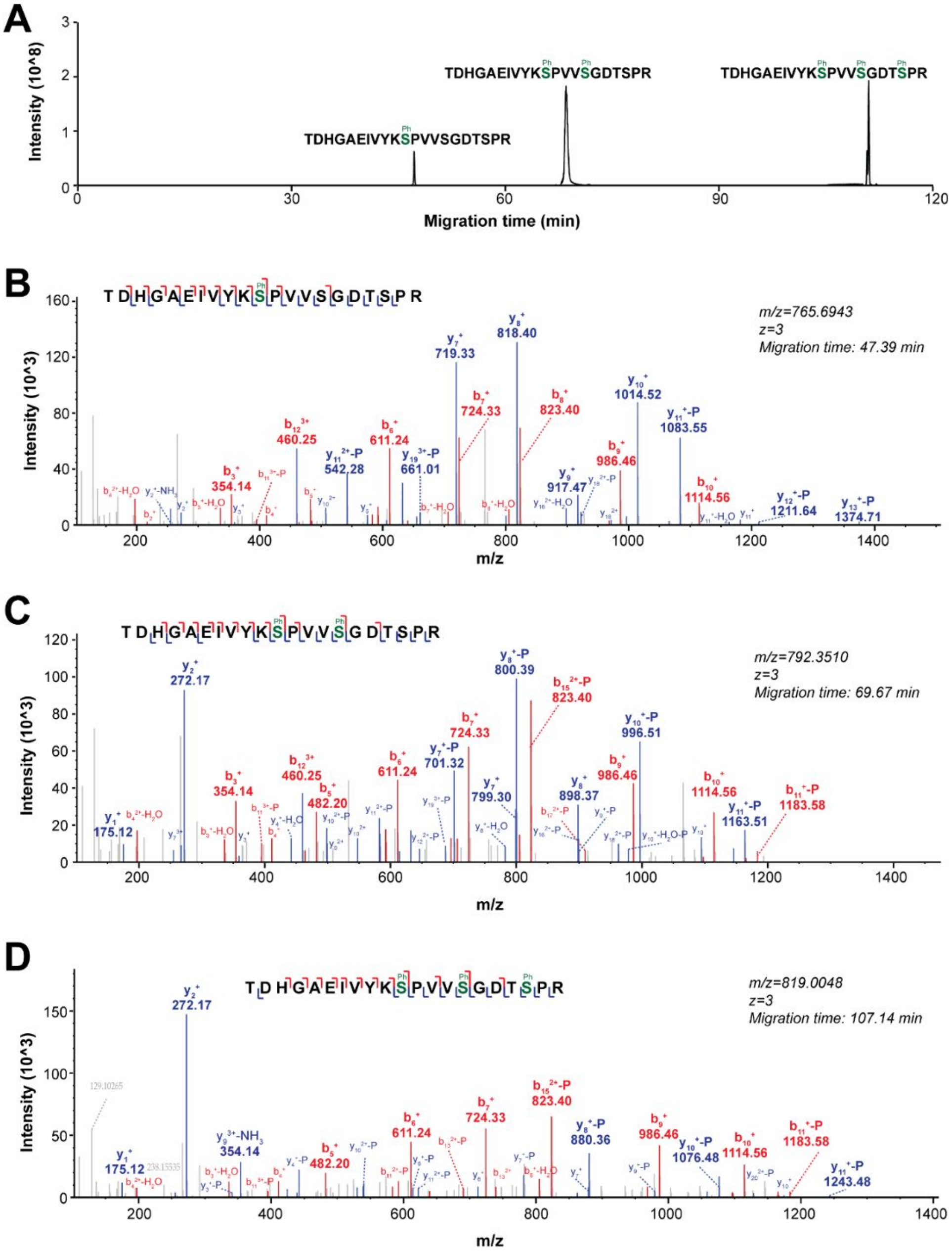

Figure S2. Example spectra of peptides carrying different numbers of phosphate groups. (A) Extracted ion electropherogram of peptide TDHGAEIVYKSPVVSGDTSPR with 1, 2, and 3 phosphorylation sites. Tandem mass spectra of peptide TDHGAEIVYKSPVVSGDTSPR carrying (B) 1, (C) 2, and (D) 3 phosphate groups.

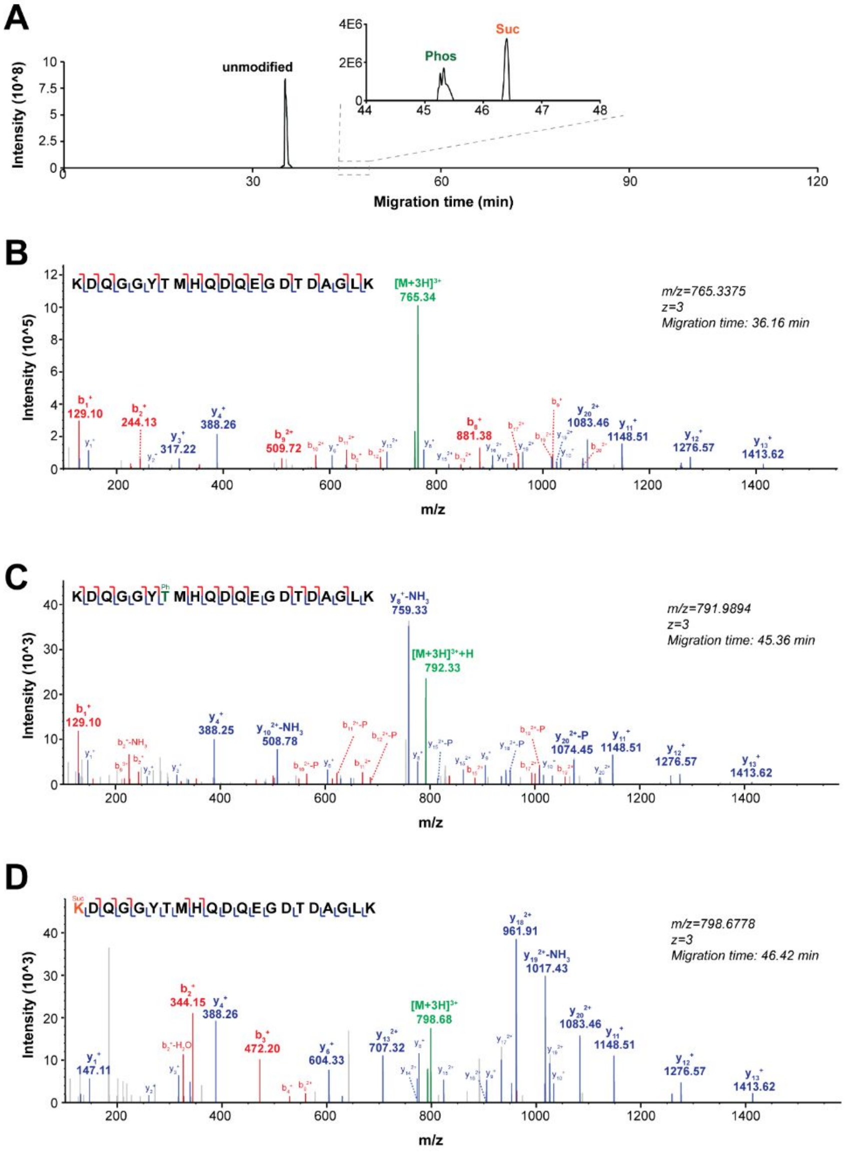

Figure S3. Example spectrum of peptide (KDQGGYTMHQDQEGDTDAGLK) without PTMs and with phosphorylation or succinylation. (A) Extracted ion electropherogram of the peptide without or with PTMs. Phos means phosphorylation and Suc indicates succinylation. (B)-(D) Annotated MS/MS spectra of the unmodified peptide (B), the phosphorylated peptide (C), and the succinylated peptide (D).

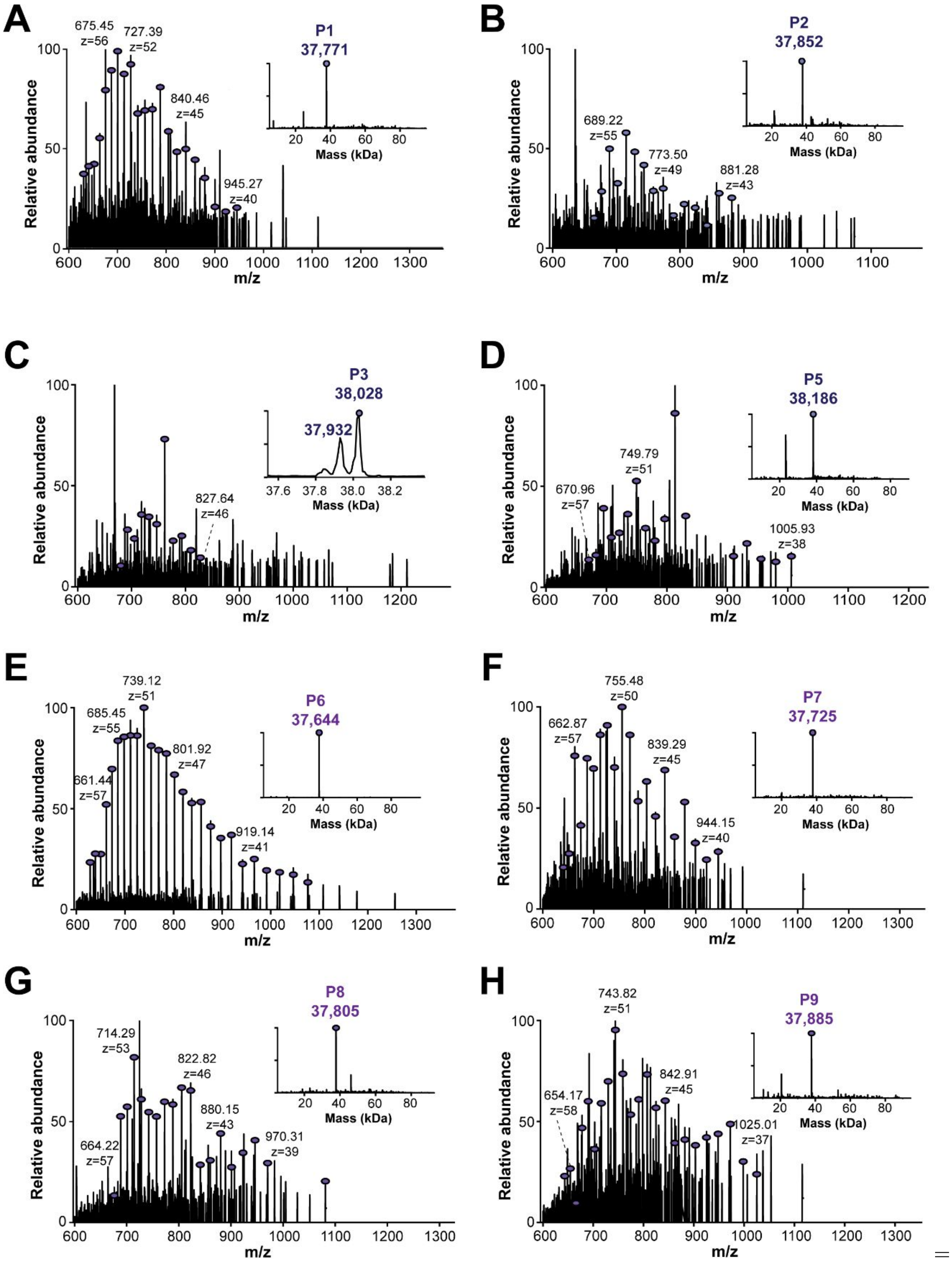

Figure S4. cIEF-MS analysis of human *p-tau-0N3R* under denaturing conditions. Mass spectra and deconvoluted masses of the proteoforms detected in P1 (A), P2 (B), P3 (C), P5 (D), P6 (E), P7 (F), P8 (G), and P9 (H).

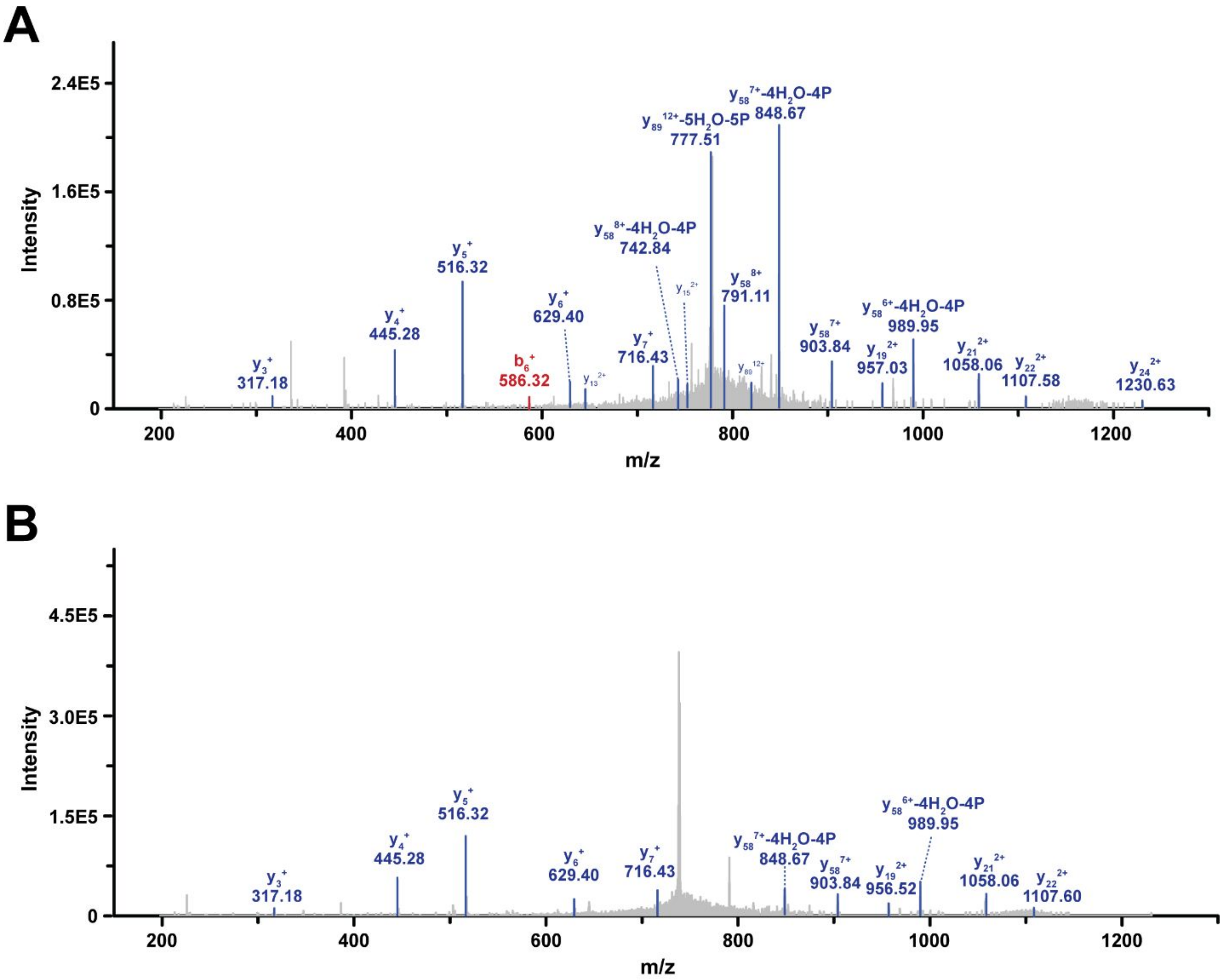

Figure S5. Annotated MS/MS spectra of two human *p-tau-0N3R* proteoforms manually in P4 (A) and P6 (B) carrying different numbers of phosphate groups under denaturing conditions.

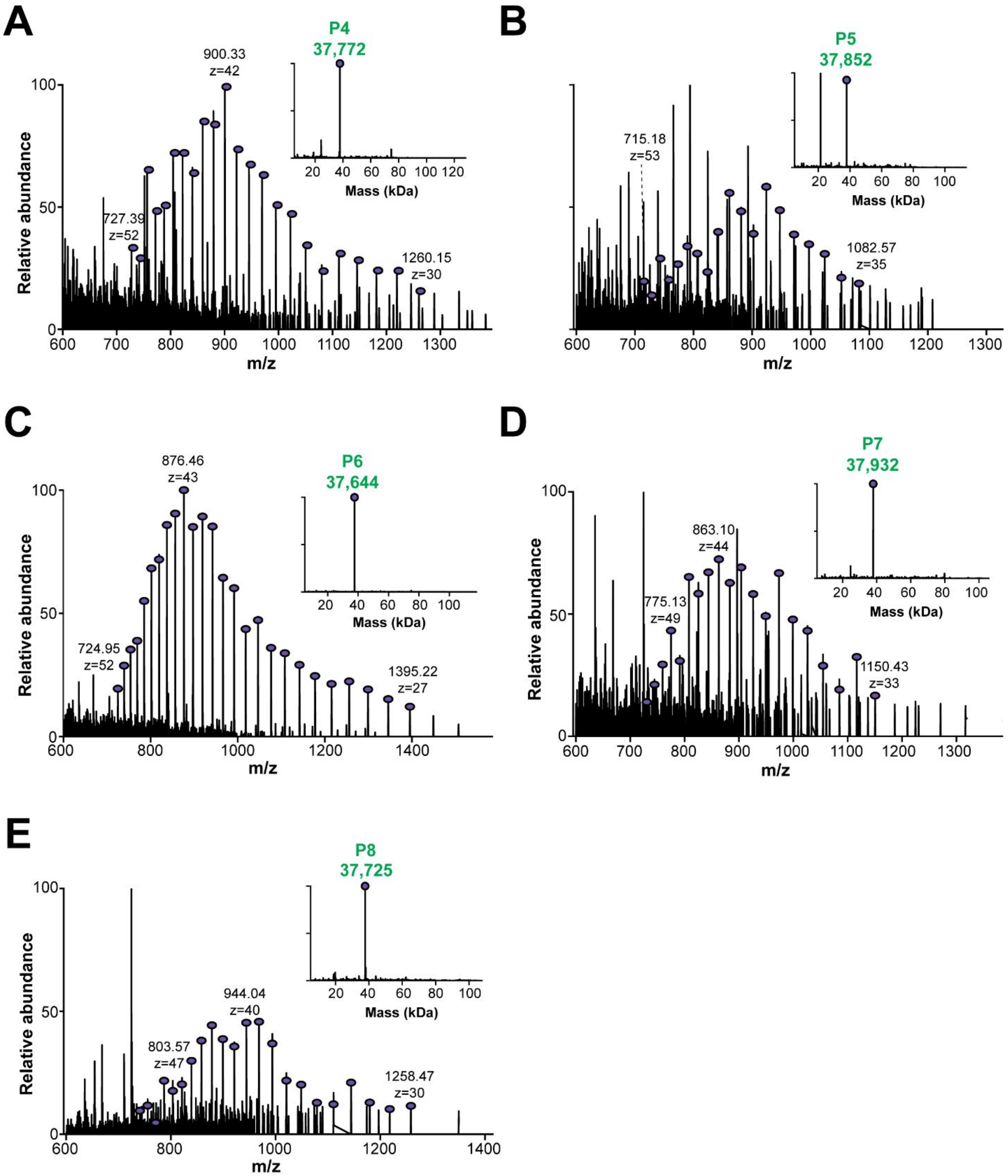

Figure S6. cIEF-MS analysis of human *p-tau-0N3R* under pseudo-native condition. (A-E) Averaged mass spectra and deconvoluted masses (inserted figures) of p-tau proteoforms detected in P4-P8.
